# Editorial: Community series: Spanish Psycholinguistics, volume II

**DOI:** 10.3389/fpsyg.2025.1718263

**Published:** 2025-11-19

**Authors:** Mikel Santesteban, Isabel Fraga, Jon Andoni Duñabeitia, Pilar Ferré

**Affiliations:** 1 Department of Linguistics and Basque Studies, University of the Basque Country (UPV/EHU), Vitoria-Gasteiz, Spain; 2 Instituto de Psicoloxía (IPsiUS), Departamento de Psicoloxía Social, Básica e Metodoloxía, Universidade de Santiago de Compostela, Santiago de Compostela, Spain; 3 Centro de Investigación Nebrija en Cognición, Facultad de Lenguas y Educación, Nebrija University, Madrid, Spain; 4 Departamento de Psicología, Rovira I Virgili University, Tarragona, Spain

**Keywords:** Spanish Psycholinguistics, Spanish psychology, psycholinguistics, cognition and language, language processing

This volume brings together a remarkable set of contributions that reflects the rich and healthy Spanish Psycholinguistics scientific community, as revealed in preceding collections (Duñabeitia et al., 2022, 2024). This second volume comprises research articles, data reports, and a data analysis package. The current studies contribute to the field of language, cognition, and communication, tackling important scientific questions about the interaction between environment and language, cross-linguistic effects in translation, lexical access effects in false memories, or syntactic disambiguation preferences during language processing. They provide psycholinguistic datasets and analysis packages of paramount importance for future psycholinguistic research in both healthy individuals and individuals with language pathologies or disorders. The studies span diverse methodological approaches and applications in Spanish, Galician, and Basque. From virtual reality experiments on reading behavior to picture and computational datasets for underrepresented languages, these works collectively highlight the dynamism and interdisciplinarity that characterize contemporary psycholinguistic and cognitive research in Spain and in the world.

The study by Rocabado et al. uses novel Virtual Reality (VR) settings to shed light on how reading—a core cognitive activity—unfolds in interaction with environmental conditions. Using immersive VR, the authors explored how visual contrast and weather scenarios (sunny vs. rainy) modulated reading. Lexical decision tasks revealed that higher visual contrast, particularly under sunny conditions, enhanced single-word recognition. Interestingly, when participants read sentences, the effect shifted: sunny weather fostered faster reading, while rainy conditions increased fixation times, suggesting deeper or more effortful processing. The work reveals that reading can be modulated by environmental weather factors, and underlines the potential of VR to simulate controlled yet ecologically valid lifelike environments in psycholinguistic research.

De Pedis et al. revisit one of psycholinguistics' classic debates: the attachment resolution of relative clauses. Testing the Pseudorelative-First Hypothesis, they conducted self-paced reading experiments in Spanish and found a consistent preference for high attachment, irrespective of pseudorelative availability. This challenges existing accounts and underscores the complexity of cross-linguistic variation in sentence processing, reminding us that no single explanation has yet fully captured the factors driving attachment preferences across different languages.

Liu's corpus study of Chinese-Spanish sight translation errors addresses an area that has received scant attention: the challenges learners face in navigating two typologically distant languages. By classifying nearly 3,000 errors across lexical, syntactic, and grammatical dimensions, and identifying substitution as the most common manifestation, the study reveals not only the pitfalls of this task but also the underlying mechanisms—from linguistic differences to cognitive load—that shape translation performance. The corpus thus lays a solid foundation for improving interpreter training and for theorizing cross-linguistic transfer effects.

In the domain of communication and accessibility, Díez et al. analyzed 1,525 pictograms from the ARASAAC platform, assessing their transparency (guessability without context) and translucency (perceived fit when labeled) in Spanish. With over 500 participants, they revealed relatively low transparency but high translucency, especially for nouns. Importantly, imageability and concreteness emerged as strong predictors of both indices. These findings are not only theoretically informative but also practically impactful, as they provide a data-driven foundation for enhancing augmentative and alternative communication systems, ensuring that pictographic tools remain accessible, intuitive, and effective for diverse users.

Álvarez de la Granja et al. expanded the MultiPic dataset to Galician, providing standardized picture norms for this language, which is spoken in Galicia, a north-western region of Spain, as well as in some border zones in the regions of Asturias and Castile and León. Their work illustrates the richness of lexical variation in Galician, shaped by regional diversity and cross-linguistic contact with Spanish. While challenges remain in terms of representativeness and modality, the Galician MultiPic offers a crucial resource for cross-linguistic and bilingual studies, and more broadly, for ensuring high-quality psycholinguistic research in minority languages.

Goikoetxea et al. address a long-standing gap by providing a computationally grounded dataset of semantic similarity for noun pairs in both Basque and Spanish. Their work integrates psycholinguistic features such as concreteness, frequency, and neighborhood density with computational measures derived from corpora and WordNet. The result is a resource that enables more precise modeling of lexical processing in two languages with rich morphology and, in the case of Basque, a relatively scarce tradition of computational resources. Beyond its immediate use in psycholinguistics, this dataset creates opportunities for cross-linguistic comparison and for strengthening the interface between natural language processing and cognitive science.

Relatedly, Benítez and Alonso provide a database on theme identifiability indices for *ad hoc* categorical lists in Spanish. Unlike associative lists, which provide clearer thematic anchors and reduce false memories, *ad hoc* categories exhibit variability that complicates retrieval. Their dataset highlights the nuanced dynamics of memory processes in contexts that more closely resemble real-world categorization, and it invites further investigation into how theme identifiability interacts with false memory formation.

Finally, a major step forward in methodological support comes from Gutiérrez-Cordero and García-Orza, who introduce *sunflower*, an open-source R package designed to streamline the analysis of multiple response attempts and error patterns in aphasia and related disorders. The package allows for automated classification of errors by lexicality, formal similarity, and semantic similarity, leveraging AI-based techniques like word2vec. Beyond its clinical applications, *sunflower* represents a broader trend toward open, computationally sophisticated tools that democratize data analysis and reduce researcher workload while improving reliability.

Taken together, these studies remind us of the extraordinary breadth of contemporary research on language and cognition, ranging from experimental innovations like VR-based reading tasks to documenting linguistic factors that shape processing and learning in native and non-native speakers of Spanish, as well as developing datasets and tools that empower future work in Spanish and understudied languages such as Galician and Basque. Importantly, this volume embraces the growing commitment to open science practices, whereby materials, datasets, and analytical tools are made openly available to the global research community; by sharing resources, researchers ensure their contributions transcend individual projects, nurturing a collaborative ecosystem that fuels scientific advancement. The current Research Topic showcases how Spanish Psycholinguistics continues to embrace openness, interdisciplinarity, and collaboration for the benefit of the broader scientific community.
